# De novo balanced reciprocal translocation mosaic t(1;3)(q42;q25) detected by prenatal genetic diagnosis: a fetus conceived using preimplantation genetic testing due to a t(12;14)(q22;q13) balanced paternal reciprocal translocation

**DOI:** 10.1186/s13039-021-00576-9

**Published:** 2021-12-04

**Authors:** Shaoqin Zhang, Jianjiang Zhu, Hong Qi, Limei Xu, Lirong Cai, Ran Meng

**Affiliations:** Prenatal Diagnosis Center, Beijing Haidian Maternal and Child Health Hospital, No.53 Suzhou Street, Haidian District, Beijing, 100080 People’s Republic of China

**Keywords:** Pre-implantation diagnosis, PGT-SR, Prenatal diagnosis, Balanced reciprocal translocation mosaicism

## Abstract

**Introduction:**

De novo balanced reciprocal translocations mosaicism in fetus conceived using preimplantation genetic testing from a different balanced translocation carrier parent has been rarely reported.

**Methods:**

Chromosomal microarray analysis, karyotype analysis and fluorescent in situ hybridization were performed to verify the type and heredity of the rearrangement. STR analysis was conducted to identify potential contamination and verify kinship. In addition, a local BLAST engine was performed to locate potentially homologous segments which might contribute to the translocation in breakpoints of chromosome.

**Results:**

A rare de novo balanced reciprocal translocations mosaicism mos 46,XY,t(1;3)(q42;q25)[40]/46,XY[39] was diagnosed in a fetus conceived using preimplantation genetic testing due to a 46,XY,t(12;14)(q22;q13) balanced translocation carrier father through multiplatform genetic techniques. Two of the largest continuous high homology segments were identified in chromosomal band 1q42.12 and 3q25.2. At the 21-months follow up, infant has achieved all psychomotor development milestones as well as growth within the normal reference range.

**Conclusion:**

We present a prenatal diagnosis of a rare de novo balanced reciprocal translocations mosaicism in a fetus who conceived by preimplantation genetic testing. The most reasonable driving mechanism was that a de novo mitotic error caused by nonallelic homologous recombination between 1q42.12 and 3q25.2 in a zygote within the first or early cell divisions, which results in a mosaic embryo with the variant present in a half proportion of cells.

**Supplementary Information:**

The online version contains supplementary material available at 10.1186/s13039-021-00576-9.

## Introduction

Balanced reciprocal translocations (BRT) are common structural chromosomal rearrangements with an incidence rate of approximately 1/500 ~ 1/625 in newborns [1]. Most BRT carriers have the normal phenotype but a high risk of abortion, infertility, or birth defects in offspring resulting from unbalanced gametes [2]. Some of these translocations disrupt haploinsufficient genes or their regulatory regions and result in clinical phenotypes, which are valuable in mapping disease genes and in illuminating cis-regulatory regions [3]. Balanced reciprocal translocations mosaicism (BRTM) has been rarely reported, and most reported cases have been diagnosed through cytogenetic analysis investigation prescribed by infertility, miscarriages, and/or unbalanced chromosome rearrangement in the offspring [4]. Furthermore, BRTM in lymphocyte cultures has been mostly described [5].

Preimplantation genetic testing (PGT) is performed before embryo transfer, and a small portion of cells will be aspirated for comprehensive chromosome screening to analyze embryos identified as balanced or normal for transplantation. It can be performed to screen embryos for monogenic/single gene disorders (PGT-M) and structural chromosomal rearrangements (PGT-SR). PGT was an established alternative to invasive prenatal diagnosis and as such may avoid adverse pregnancy in couples with structural chromosome abnormalities, and also for a high risk of transmitting genetic disorders [6–8]. Following PGT-SR, normal/balanced embryo transfers, pregnancy outcomes of reciprocal carriers with recurrent miscarriage have been reported to improve, with a decrease in miscarriage rates and an increase in the ongoing pregnancy rates [9, 10]. PGT-SR can hardly distinguish between balanced and normal embryos further. Consequently, an embryo may still be a carrier of BRT inherited from a parent. Here we reported a rare case of BRTM mos 46,XY,t(1;3)(q42;q25)[40]/46,XY[39] in a fetus conceived using PGT-SR in a t(12;14)(q22;q13) BRT carrier father.

## Materials

### Statement

The participants agreed to donate the remaining samples and data to scientific research, technical innovation and clinical application after the identifiable personal information was removed. The participants provided their informed consent.

### Sample information

A 31-year-old pregnant woman and her 32-year-old husband have been suffering from primary infertility for 3 years. The husband was diagnosed with asthenospermia with karyotype 46,XY,t(12;14)(q22;q13), but the wife’s chromosomes were normal. Subsequently, the couple underwent in vitro fertilization and embryo transfer and PGT-SR in other hospitals. In accordance with routine protocols, a normal/balanced embryo (aCGH or NGS are unable to distinguish between balanced and normal embryos) was transferred, resulting in a successful pregnancy.

According to the Chinese expert consensus on genetic diagnosis and screening, invasive prenatal diagnosis is required for those who achieve ongoing pregnancy after PGT embryo transfer [11]. After providing informed consent, the pregnant woman agreed to accept interventional prenatal diagnosis (amniocentesis) in the second trimester of pregnancy. Karyotype and SNP-array analyses were performed at 19 weeks of gestation in order to evaluate chromosome abnormalities. Conventional cytogenetic analysis was also conducted on the umbilical cord blood of fetal after birth. Short tandem repeat (STR) profiling has been used in paternity testing and excluding potential maternal contamination.

## Method

### Cell culture and karyotype analysis

Cell culture and G-band karyotype analysis were performed in accordance with standard cytogenetic methods. Fetal cells obtained from amniotic fluid and umbilical cord blood( after birth) were cultured with a double-line by using standard methodologies [12]. In case of suspected mosaicism, additional cells were analyzed.

### Chromosomal microarray analysis (CMA)

CMA was performed using CytoScan®750 K array(Thermo Fisher, USA) according to manufacturer’s instructions. The procedure included DNA extraction, digestion and ligation, PCR amplification, purification, fragmentation, labeling, hybridization, washing and scanning. Data was analyzed with Affymetrix® Chromosome Analysis Suite (ChAS) 4.0 Software. The threshold of CNVs was set at 100 kb with marker count ≥ 25.

### Fluorescent in situ hybridization (FISH)

Metaphase FISH on cultured amniocytes were undertaken using subtelomere probes of chromosome 1 (CEB108/T7 at 1p36.3 and VIJyRM2123 at q44), 3 (3PTEL25 at 3p26 and 3QTEL05 at q29), 12 and 14 (BlueGnome, Cambridge, UK) to reveal the reciprocal translocations. 20 metaphases were analyzed for each probe.

### STR

STR analysis was performed exclude maternal contamination and identify familial affinities by using a five-dye fluorescent technology and a co-amplification method to detect 21 loci ( 20 STR loci and amelogenin, Additional file 1: Table S1) (Microreader™ 21[Direct] ID System, Microread Genetics, China) in accordance with the operating procedure. Among the 21 STR loci, heterogenic contamination was verified when at least three loci had more than two alleles.

### BLAST engine

According to the karyotype analysis findings, we systematically evaluated the genomic sequence within the potentially breaking bands, 1q42 and 3q25 by using a local BLAST engine [13]. The intention was to locate potentially homologous segments which might contribute to the translocation.

## Result

### Cytogenetic analysis

**Fig. 1:**
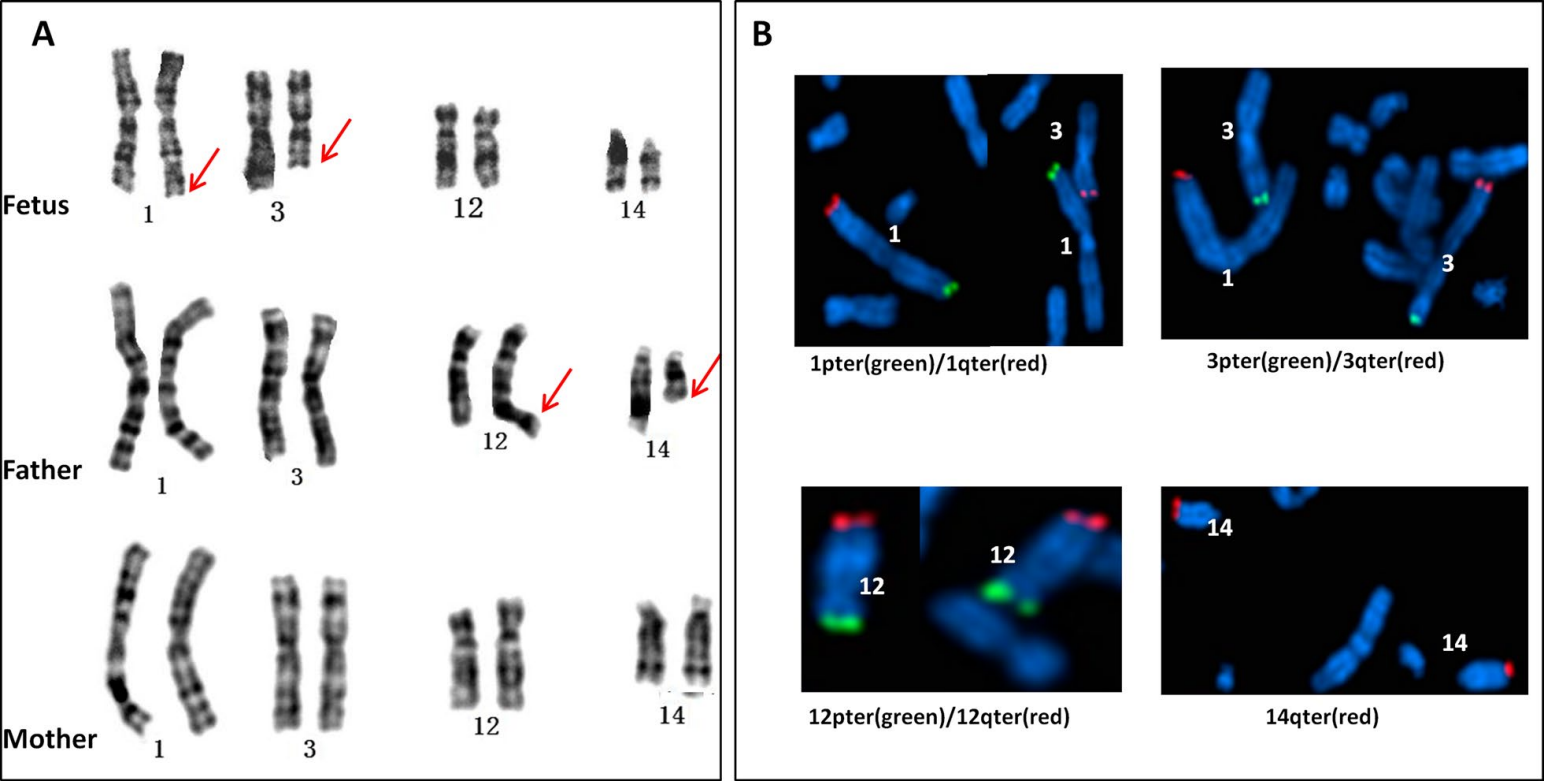
**A** Partial chromosomal karyotypes of the family by G-banding. Fetus (above): mos 46,XY,t(1;3)(q42;q25)[40]/46,XN[39]; Father(middle): 46,XY,t(12;14)(q22;q13); Mother (bottom): 46,XX. **B** Metaphase FISH on cultured amniocytes showed that the fetus was a carrier of the translocation between the subtelomere of chromosomes 1 and 3, and with the normal subtelomere probes of chromosomes 12 and 14.

The routine cytogenetic analysis of the fetus revealed a BRTM with the karyotype mos 46,XY,t(1;3)(q42;q25)[40]/46,XY[39]. Similar levels of mosaicism for the same balanced translocation were found in neonatal umbilical cord blood samples. The father's karyotype was 46,XY,t(12;14)(q22;q13), and the mother’s karyotype was normal (Fig. 1A).

### CMA

**Fig. 2:**
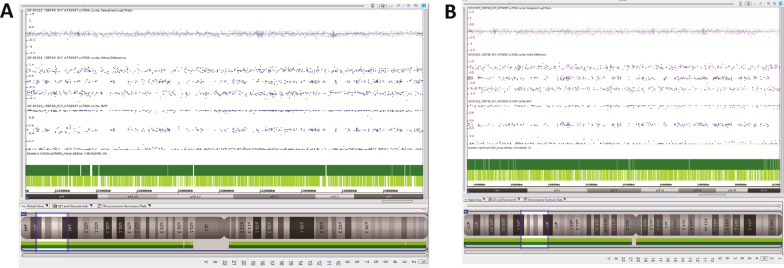
SNP-array profile of chromosome 1q42 (**A**) and chromosome 3q25 (**B**) in fetal amniotic fluid cells excluded cryptic genomic imbalances at translocation breakpoints

SNP-array profile of chromosome 1 and chromosome 3 had been shown in Fig. 2. For each profile, the lower plot shows a logR ratio of 0 at the breakpoint (1q42 and 3q25), where 0 suggests the copy number is equal to 2, so SNP-array excluded cryptic genomic imbalances at translocation breakpoints. For both subjects, normal B allele frequencies (BAF) profiles for all chromosomes demonstrate the absence of chimerism.

### FISH

FISH showed that the fetus was a carrier of the translocation between the subtelomere of chromosomes 1 and 3, with a mosaic rate of 40% (8 of 20 metaphases). However, the subtelomere probes of chromosomes 12 and 14 were normal in 20 metaphases (Fig. 1B).

### STR

**Fig. 3:**
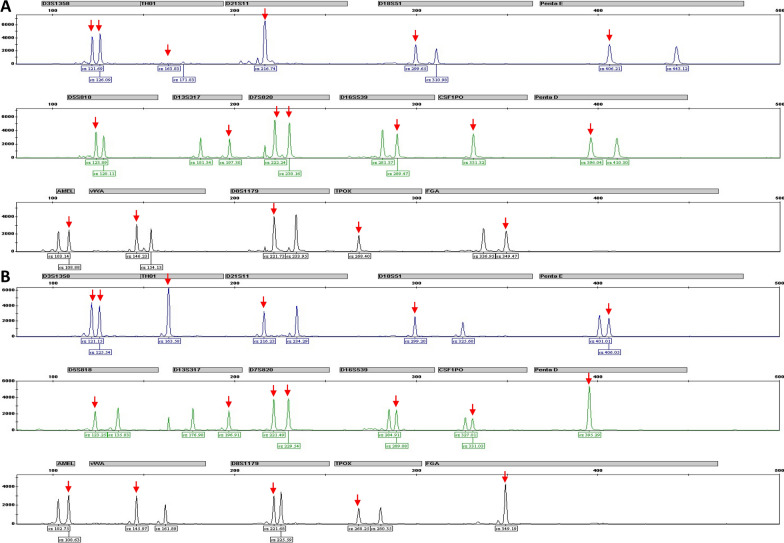
STR results showed that at least one paternal allele (**A**) could be found at each allele (**B**) in the fetus. And none of the studied loci showed more than two alleles

STR results showed that none of the studied loci showed more than two alleles, thus excluding the possibility of exogenous contamination. All the tested STR loci of the fetus genome present one of paternal marker (Marked by red arrow in Fig. 3).

According to all methods used, the fetal karyotype could be written as mos 46,XY,t(1;3)(q42;q25)[40]/46,XY[39].ish t(1;3) (VIJyRM2123-,3QTEL05 + ;3QTEL05-,VIJyRM2123 +)[8] /1p36.3q44(CEB108/T7 × 2,VIJyRM2123 × 2),3p26q29(3PTEL25 × 2,3QTEL05 × 2)[12].arr (X,Y) × 1,(1–22) × 2.

### BLAST engine

**Fig. 4:**
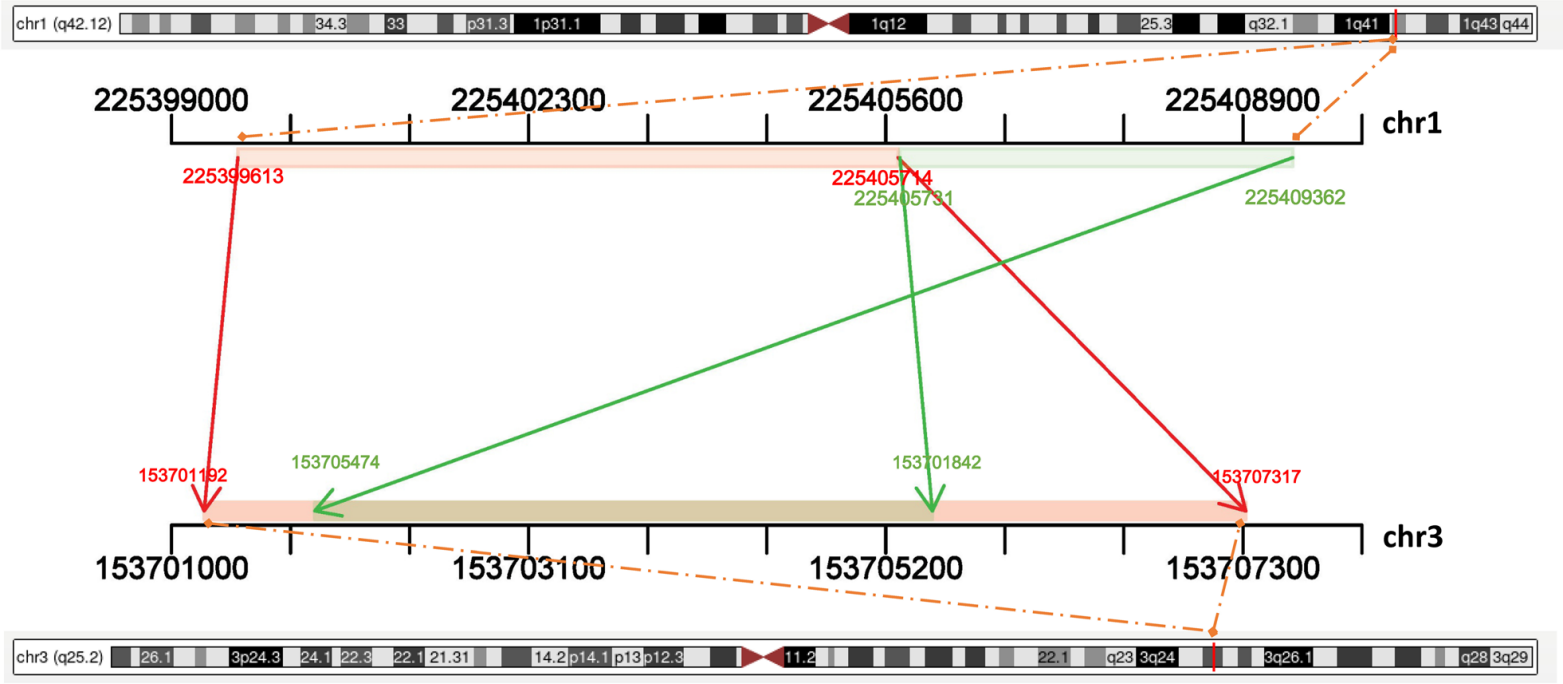
Mapping of homologous chromosomal regions within 1q42.12 and 3q25.2 by a local BLAST engine

Using a local BLAST engine, we identified two of the largest continuous segments masked with lowercase nucleotide bases, while showing high similarity between the two chromosome regions according to hg19 reference genome sequence. The percentage of homologous sequences in the two fragments on chr1 and chr3 was 90.49% and 86.26%, respectively (Table 1, Fig. 4).

**Table 1:** Candidate chromosomal regions within the two breaking bands, one row for each pair of homologous alignment

From Chr	Start(bp)	End(bp)	To Chr	Start(bp)	End(bp)	Homology (%)
chr1	225399613	225405714	chr3	153701192	153707,317	90.49
chr1	225405731	225409362	chr3	153705474	153701842	86.26

### Follow-up results of fetal

Ultrasound imaging revealed normal fetal development at 30 weeks of gestation. However, at 31 weeks and 2 days of gestation, premature delivery was caused by uncontrollable uterine contractions. The birth weight of the newborn was 1680 g and the length was 42 cm. An Apgar score of 10 was obtained at 1, 5 and 10 min. Neonatal echocardiography was normal except for the patent foramen ovale. Placental pathology showed no idiopathic abnormality.

During the 21st month of follow-up, the growth and development of the infant were normal, he raised his head, turned over and sat on schedule. At 18 months, he walked steadily. Now he walks runs and jumps freely. According to his parents' description, his language ability is better than that of his peers.

## Discussion

De novo apparently BRT is detected in approximately 1/800 ~ 1/1000prenatal tests [14, 15]. Chromosomes 22, 7, 21, 3, 9 and 11 are preferentially involved, whereas chromosomes X, 19, 12, 6 and 1are rarely implicated. Breakpoints are nonrandomly distributed across chromosomes. The location of recurrent breakpoints is associated with fragile sites in chromosomes 11, 7, 10 and 22, but this relationship is not observed in chromosome 3 [15]. In the present case, the break points were confirmed at 1q42 and 3q25. This finding partly was consistent with the involved chromosome described in previous studies, but different breakpoints were detected.

BRTM has been rarely reported [4] and mainly observed in subjects with a normal phenotype accompanied by reproductive failure. Opheim et al. [16] estimated that the frequencies of BRTM in postnatal and prenatal populations are 5.7 × 10^−5^ and 4.1 × 10^−5^, respectively. Recently, Garzo et al. [4] noted that only 25 cases were previously reported, and described 10 new cases of BRTM. They suggested that carrier individuals may be more frequent than expected. However, the incidence of BRTM is still poorly defined, may be due to the missed or inaccurate diagnosis of BRTM during the detection, such as low proportion mosaicism, and lack of technical means to detect micro-abnormalities in chromosome. Lastly, the size of recombination fragments, the resolution of chromosome bands, and the number of cell counts are all related to the accurate diagnostic of BRTM. Because SNP-array (copy number + SNP arrays) detect copy-number changes and allele genotypes in a single platform, they can provide an internal confirmation of CNVs that may eliminate the need for secondary confirmatory testing such as quantitative PCR, multiplex ligation-dependent probe amplification, or FISH [17]. Furthermore, SNP-array analysis ruled out chimerism as the pathomechanism of BRTM for our case because of the absence of new genotypes across all chromosomes. Indeed, mosaicisms must be confirmed in at least two different cultures or in various tissues to exclude the possibility of the in vitro origin of chromosomal rearrangement [18]. In our study, similar mosaicism levels for the same balanced translocation were found in the amniotic fluid and cord blood. This result confirmed that the proposita has true BRTM and that the cytogenetic findings are not an artefact.

Although the origin of BRTM is still obscure, the plausible driving mechanism has been postulated to be either postzygotic [19] or prezygotic [20]. Postzygotic events have two hypothetical mechanisms: mosaicisms (which occurs during the mitosis of single zygotes) and chimerism [21] (which is the fusion of two zygotes). Chimerism can be distinguished from mosaicism by evaluation of the extent of genotypic differences, such as STR. Indeed, in mosaicism one paternal allele and one maternal allele should be found at all loci, whereas in chimerism two alleles for one or both parental contributions should be observed in at least at one locus [21]. The apparent STR result showed that one paternal allele was found at all loci (Fig. 3). Considering that the mosaicisms proportion of our case was close to 50% in amniotic fluid cells and umbilical cord blood, the mechanism of BRTM in our case was plausible because a de novo mitotic error might originate from a zygote during the first or early cell divisions; this error likely resulted in a mosaic embryo with the variant present in a half proportion of cells, and this mosaicism can affect somatic and/or gonadal tissues [22]. However, the mosaic ratio of different fetal tissues might vary because of the growth deviation of different cell types during cell culture.

Most constitutional genomic rearrangements are created through 1 of the 4 well-known mechanisms, i.e., nonallelic homologous recombination, erroneous repair after double-strand DNA breaks, replication errors, and retrotransposition [23]. According to the karyotyping analysis findings, we systematically evaluated the genomic sequence within the potentially breaking bands, 1q42 and 3q25. The intention was to locate potentially homologous segments which might contribute to the translocation. Using a local BLAST engine, we identified two of the largest continuous segments masked with lowercase nucleotide bases, while showing high similarity between 1q42.12 and 3q35.2 regions according to hg19 reference genome sequence. These two connecting segments on 1q42.12 are placed linearly with a small overlap. Interestingly, the smaller segment is reversely mapped within the bigger corresponding segment on 3q25.2, suggesting their complex inter-chromosomal rearrangement potential. However, we did not directly validate the candidate regions for breakpoints. Therefore, further research is still needed.

The relationship between phenotype and BRT mosaicism/chimerism (including tissue-specific mosaicism) is unclear. A long-term follow-up study has suggested that children with prenatally diagnosed de novo apparently BRT have similar long-term health and developmental outcomes to those of children of the same age in a general population [24]. However, a de novo apparently balanced translocation may still lead to the disruption of a gene and cause abnormal phenotypic consequences [25, 26]. No significant abnormality in prenatal ultrasound and postpartum physical examination other than premature delivery and low birth weight was found in our case. During the 21st month of follow-up, the infant achieved all psychomotor developmental milestones and growth within the normal reference range. Certainly, he needs long-term health and developmental follow-up. When he reaches the child-bearing age, sperm karyotype analysis can be applied to determine the rate of gonadal mosaicismin and guide his fertility. Assisted reproductive technology will be recommended to avoid adverse pregnancy if necessary.

## Conclusion

To the best of our knowledge, only Kim et al. [27] reported the first case of a de novo BRT conceived using PGT from a balanced translocation carrier mother similar to our patient. Thus, our case is the second unique case reported in the literature for prenatal diagnosis of a de novo BRTM mos 46,XY,t(1;3)(q42;q25)[40]/46,XY[39] in a fetus conceived via PGT-SR from a t(12;14)(q22;q13) balanced translocation carrier father. In our case, the most reasonable driving mechanism of BRTM was that a de novo mitotic error caused by nonallelic homologous recombination between 1q42.12 and 3q25.2 in a zygote within the first or early cell divisions, which results in a mosaic embryo with the variant present in a half proportion of cells. However, further studies should be performed to determine if the de novo BRT is an accidental event or if PGT induces cell damage leading to new translocation.

## Supplementary Information

**Additional file 1. Supplemental Table 1**. The specific STR loci informations of Microreader™ 21(Direct) ID System.

## Data Availability

All data generated or analyzed in this study are included in this published article.

## Abbreviations

PGT: Preimplantation genetic testing
BRTM: Balanced reciprocal translocations mosaicism
PGT-SR: PGT for structural rearrangements
CNVs: Copy number variants
