# Supplementary material for: De novo balanced reciprocal translocation mosaic t(1;3)(q42;q25) detected by prenatal genetic diagnosis: a fetus conceived using preimplantation genetic testing due to a t(12;14)(q22;q13) balanced paternal reciprocal translocation
Source: Mol Cytogenet. 2021 Dec 4;14:55. doi: 10.1186/s13039-021-00576-9 (PMC8645079; doi:10.1186/s13039-021-00576-9)
Supplement: Supplementary file 1 — Additional file 1. Supplemental Table 1. The specific STR loci informations of Microreader™ 21(Direct) ID System. [file 13039_2021_576_MOESM1_ESM.docx]

**Supplemental Table 1**.The specific STR loci informations of MicroreaderTM 21(Direct) ID System

| **loci** | **tag** | **location** | **Fragment size** | **loci** | **location** | **tag** | **Fragment size** |
| --- | --- | --- | --- | --- | --- | --- | --- |
| D19S433 | AFM | 19q12 | 88-135. | Amel | X/Y | HEX | 95-107. |
| D5S818 |  | 5q23.2 | 136-189. | D3S1358 | 3p21.31 |  | 115-160. |
| D21S11 |  | 21q21.1 | 193-269. | D13S317 | 13q31.1 |  | 168-207. |
| D18S51 |  | 18q21.33 | 271-366. | D7S820 | 7q21.11 |  | 210-255. |
| D6S1043 |  | 6q15 | 370-445. | D16S539 | 16q24.1 |  | 257-310. |
|  |  |  |  | CSF1PO | 5q32 |  | 312-366. |
|  |  |  |  | Penta D | 21q22.3 |  | 364-445. |
| **loci** | **tag** |  | **Fragment size** | **loci** |  | **tag** | **Fragment size** |
| D2S441 | TAMRA | 2p14 | 75-117. | TH01 | 11p15.5 | ROX | 92-138. |
| vWA |  | 12p13.31 | 118-190. | D12S391 | 12p13.2 |  | 141-205. |
| D8S1179 |  | 8q24.13 | 197-259. | D2S1338 | 2q35 |  | 209-275. |
| TPOX |  | 2p25.3 | 261-306. | FGA | 4q31.3 |  | 277-444. |
| Penta E |  | 15q26.2 | 308-433. |  |  |  |  |
